# The expression and clinical significance of matrix metalloproteinase 7 and tissue inhibitor of matrix metalloproteinases 2 in clear cell renal cell carcinoma

**DOI:** 10.3892/etm.2012.859

**Published:** 2012-12-17

**Authors:** HONGSHENG LU, ZHAOHUI YANG, HUI ZHANG, MEIFU GAN, TAO ZHOU, SILING WANG

**Affiliations:** 1 Department of Pathology, Taizhou Central Hospital of Taizhou Enze Medical Group, Taizhou 318000;; 2 Department of Pathology, Taizhou Hospital of Taizhou Enze Medical Group, Linhai 317000, P.R. China

**Keywords:** clear cell renal cell carcinoma, matrix metalloproteinase 7, tissue inhibitor of matrix metalloproteinases 2, prognosis, tissue microarray

## Abstract

Renal cell carcinoma (RCC) is one of the most common malignant neoplasms in the urinary system, and has a high frequency of local invasion and distant metastasis. This study assessed the expression and clinical significance of matrix metalloproteinase 7 (MMP-7) and tissue inhibitor of matrix metalloproteinases 2 (TIMP-2) in human clear cell renal cell carcinoma (CCRCC) by tissue microarray, immunohistochemistry and RT-PCR analysis. The expression of MMP-7 in CCRCC tissues was significantly higher compared with that in the control group (CG) and TIMP-2 expression in CCRCC, by contrast, was lower compared with that in the CG. The expression of MMP-7 and TIMP-2 in the CCRCC tissues was significantly correlated with the pathological grade and clinical stage. Log-rank analyses indicated that upregulation of MMP-7 and downregulation of TIMP-2 expression may occur during the progression of CCRCC, and Cox multivariate survival rate analysis demonstrated that there was also a positive correlation between the pathological grade, clinical stage, MMP-7 expression and survival rate. Thus, MMP-7 is an independent prognostic factor and MMP-7 and TIMP-2 may serve as useful molecular markers for evaluating prognosis in CCRCC patients.

## Introduction

Renal carcinoma is the most common malignant tumor of the kidney and accounts for ∼3% of all malignant tumors and 80% of malignant kidney tumors. Among renal carcinomas, 79% are classified as clear cell renal cell carcinomas (CCRCC) (1). However, little is known about the molecular and cellular mechanisms involved in the development of CCRCC.

Although there are various factors associated with malignant aggressiveness, destruction of the extracellular matrix (ECM) is one of the major early steps in a number of malignant tumors. A major family of ECM-degrading enzymes involved in this process is the matrix metalloproteinase (MMP) family, which plays important roles in cancer invasion and metastasis (2,3). In addition to their invasive function, MMPs are also associated with cell proliferation and angiogenesis in various cancers (4–6). Based on these features, a number of researchers are considering the pathological significance of MMPs in cancer cells and the potential of MMP inhibitors in the antitumor treatment of various malignancies. Tissue inhibitors of matrix metalloproteinases (TIMPs) are known to have the ability to inhibit the catalytic activity of MMPs. It is thought that the balance between MMPs and TIMPs determines the proteolytic activity *in vivo*(7,8). The ratio of MMPs to TIMPs, which is required to be close to 1 to neutralize enzymatic activity, means that small changes in MMP and TIMP levels lead to biologically significant changes in net proteolytic activity. If MMP expression increases and/or TIMP expression decreases, the balance is greatly affected (9).

The expression of MMP-1, -2, -3, -9, -10 and -11, as well as TIMP-1 and -2, has been analyzed in CCRCC (10–17). However, little or no information concerning the association of MMP with TIMP in human CCRCC tissues and the clinicopathological significance of such expression on survival rate has been reported. Moreover, the correlation between the balance of MMPs and TIMPs in CCRCC and the clinicopathological characteristics and survival rate using tissue microarrays have not been reported. In the present study, we focused on the role of MMP-7 associated with TIMP-2 in human CCRCC tissues to determine the correlation with clinicopathological features and survival rate using tissue microarray, immunohistochemistry and RT-PCR to evaluate the clinical value of MMP-7 and TIMP-2 proteins in CCRCC.

## Materials and methods

### Materials and tissue microarray

Subjects diagnosed with CCRCC based on pathological examination of patient tissues following radical surgery at Taizhou Central Hospital of Taizhou Enze Medical Group and Taizhou Hospital of Taizhou Enze Medical Group between January 1997 and December 2006 were selected. The patient population included 63 men and 35 women, with an average age of 55.16±10.40 years (range, 25–83 years). Cases were graded based on the 2004 World Health Organization (WHO) pathological Fuhrman nuclear grading standards (18). In all, 47 cases were classified as grade I, 39 cases as grade II, 8 cases as grade III and 4 cases as grade IV. According to the 2004 WHO clinical staging standards, 61 CCRCC patients were stage I, 24 were stage II, 8 were stage III and 5 were stage IV.

Histopathological examination and immunohistochemical staining were performed using cancer tissues from the 98 CCRCC patients enrolled in the study. Paraffin-embedded CCRCC tissues (98 cases) and normal renal tissues (28 cases) were retrieved and tissue microarray slides were constructed according to a previously published method (19). The micro-array contained 126 cases in total, including CCRCC and control group (CG) specimens. This study was approved by the Taizhou Enze Medical Group Research Ethics Committee. All patients provided written informed consent in order to participate in this study.

### Immunohistochemistry

Tissue microarray sections were dewaxed in xylene, rehydrated in alcohol and immersed in 3% hydrogen peroxide for 10 min to suppress endogenous peroxidase activity. Antigen retrieval was performed by heating (100°C) each section for 30 min in 0.01 mol/l sodium citrate buffer (pH 6.0). After three rinses (each for 5 min) in phosphate-buffered saline (PBS), sections were incubated for 2 h at room temperature with a mouse anti-human MMP-7 antibody (Dako, Carpinteria, CA, USA; 1:100) or mouse anti-human TIMP-2 antibody (Dako; 1:100) diluted in PBS. After three washes (each for 5 min) in PBS, sections were incubated with horseradish peroxidase-labeled goat anti-mouse immunoglobulin (Dako) for 1 h at room temperature. After three additional washes, peroxidase activity was developed with diaminobenzidine (DAB) at room temperature. EnVision staining was performed. PBS was substituted for the primary antibody as a negative control and the known positive slips served as positive controls.

The positive staining of the MMP-7 and TIMP-2 expression were mainly located in the cytoplasm with brown-yellow granules. In each section, 5 high-power visual fields were randomly selected and observed. Two hundred cells in each visual field were counted. The staining was judged according to the percentage of positive cells: <5% positive cells was negative (-); 5–20% positive cells was weak positive (+); 20–50% positive cells was middle positive (++) and >50% positive cells was strong positive (+++).

### RT-PCR

RT-PCR analysis was performed to determine the mRNA levels for the last 61 CCRCC and 22 CG patients. For these analyses, fresh renal tumor tissue and normal renal tissue were harvested during the surgical resection of the tumor and immediately frozen in liquid nitrogen and stored in a −70°C ultra-low temperature freezer.

Three pairs of primers for MMP-7, TIMP-2 and GAPDH (reference gene) were designed using the computer-assisted software Primer Premier 5.0. Gene sequences from Genbank were used in the primer design. These primers are summarized in Table I. The specificity of these primers was confirmed using the BLAST system. The 3 pairs of primers were synthesized by Shanghai Sangon Bioengineering Co. Ltd. (Shanghai, China).

Total RNA was isolated using TRIzol and the RNA purity and concentration were determined based on A260 and A280 absorption. The RT-PCR procedure was performed as follows: i) reverse transcription was carried out using 2 *μ*g RNA in a mixture containing 100 ng (2 *μ*l) random primers and 15 *μ*l water. The mixture was heated at 65°C for 5 min and then cooled to 4°C. This was followed by the addition of 2.0 *μ*l diethylpyrocarbonate (DEPC) water, 5.0 *μ*l 5X RT-buffer, 1.25 *μ*l 10 mmol/l deoxyribonucleotide triphosphate (dNTP), 0.75 *μ*l 50 U/*μ*l RNAs and 1.0 *μ*l Moloney murine leukemia virus (MMLV; 10 U/*μ*l). The mixture was heated at 37°C for 60 min and cooled to 4°C for storage. ii) PCR amplification was performed in a 25-*μ*l PCR reaction containing 2.5 *μ*l 15 mmol/l MgCl_2_, 0.5 *μ*l 10 mmol/l dNTP, 1 *μ*l 1.5 units Taq DNA polymerase, 1 *μ*l 3′ and 5′ primers and 2.5 *μ*l template DNA. The amplification conditions were different for each target gene. MMP-7 and TIMP-2 were amplified for 30 cycles, each including a predenaturation step at 95°C for 10 min, a denaturation step at 95°C for 60 sec, a renaturation step 52°C for 30 sec and an extension step at 72°C for 45 sec. During the last cycle, the 72°C step was held for 5 min. GAPDH was amplified for 30 cycles, each consisting of predenaturation at 95°C for 10 min, denaturation at 94°C for 60 sec, renaturation at 60°C for 45 sec and extension at 72°C for 60 sec. During the last cycle, the 72°C step was held for 5 min.

For detection of the amplification products, 10 liters of PCR product and 2 liters of loading buffer were separated on a 2% agarose electrophoresis gel, run at 100 V for 25 min and the optical density of target bands was analyzed using a gel analysis system. The ratio of MMP-7 or TIMP-2 band optical density to GAPDH band optical density was calculated.

### Follow-up

The follow-up was carried out by telephone or letter. The survival time was defined as the time from diagnosis to mortality or to the final examination. There were 74 cases that were followed up for >5 years, including 58 cases of survival, 18 cases of mortality due to tumor recurrence and/or metastasis, 2 cases of mortality as a result of another disease and 6 cases which were lost to follow-up.

### Statistical analysis

The SPSS 10.0 (SPSS Inc., Chicago, IL, USA) statistical software package was used to analyze the data. Chi-square (χ^2^) tests were used for results presented as percentages, t-tests were used for results with means belonging to a normal distribution and Spearman grade-relevance analysis was used to determine the correlation between two variables. Postoperative survival rates were estimated using a lifespan table. Survival curves were created using the Kaplan-Meier method and analyzed by log-rank test. In addition, the Kaplan-Meier method and log-rank test were used for single variant analysis and a Cox model was used to analyze correlations between survival rates and multiple variables. All statistical tests were two-sided and P<0.05 was considered to indicate a statistically significant difference.

## Results

### MMP-7 expression in CCRCC and normal kidney with immunohistochemical staining

**Figure 1.**
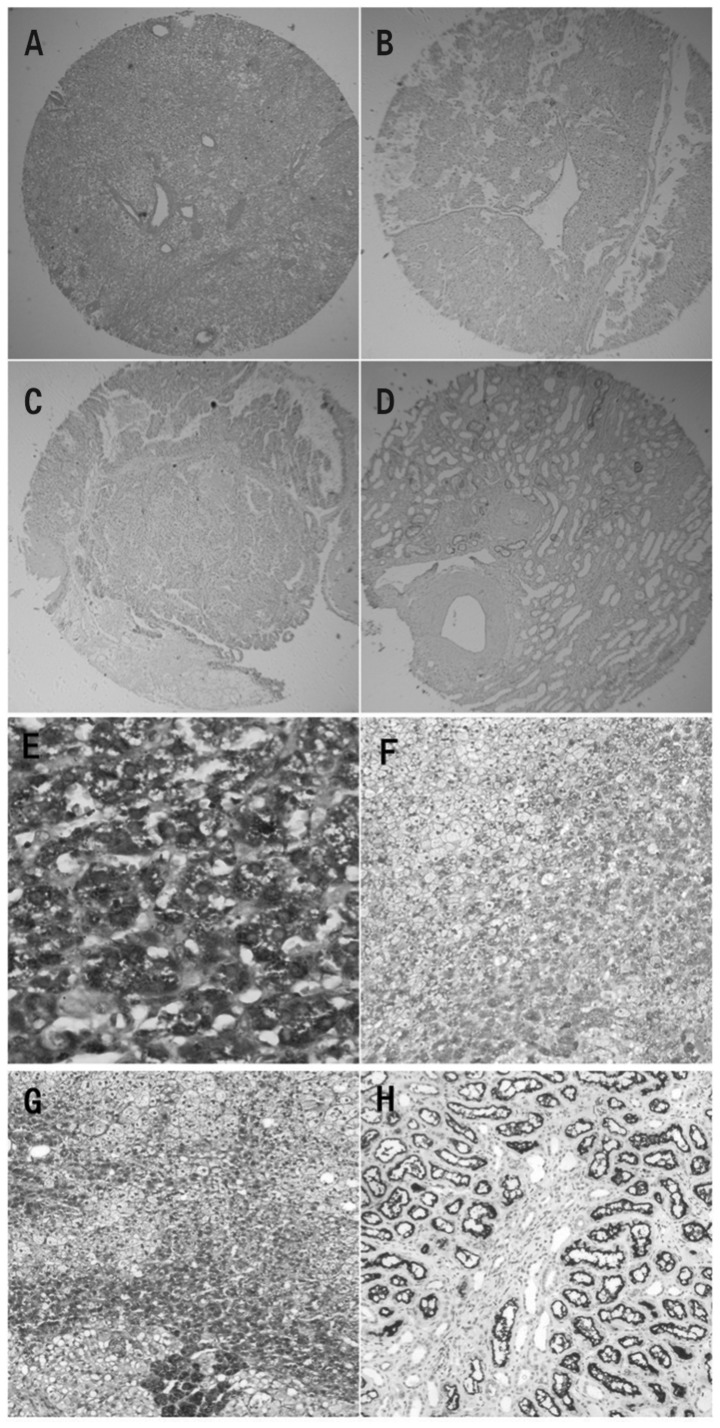
Expression of MMP-7 and TIMP-2 in CCRCC and normal tissues in a tissue microarray. (A) H&E staining of CCRCC (original magnification, ×10). (B) IHC staining for MMP-7 expression in CCRCC (original magnification, ×10). (C) IHC staining for TIMP-2 expression in CCRCC (original magnification, ×10). (D) TIMP-2 expression was restricted to the tubular structures in normal renal tissues (original magnification, ×10). (E) IHC staining for MMP-7 protein revealed that MMP-7 was situated mainly in the cytoplasm and on the membrane in stage IV (original magnification, ×400). (F) MMP-7 was moderately expressed in grade II (original magnification, 100×). (G) TIMP-2 expression was strong in grade I (original magnification, ×100). (H) Levels of TIMP-2 expression in renal tubule epithelial cells (original magnification, ×100). MMP-7, matrix metalloproteinase 7; TIMP-2 tissue inhibitor of matrix metalloproteinases 2; CCRCC, clear cell renal cell carcinoma; H&E, hematoxylin and eosin; IHC, immunohistochemistry.

Representative sections of CCRCC kidney tissue stained with hematoxylin and eosin and with immunohistochemical staining for MMP-7 are shown in Fig. 1A and B, respectively. In the CCRCC kidney tissues, the cytoplasm of cancerous cells presented positive staining for MMP-7, with a positive expression rate of 62.2% (61/98). By contrast, MMP-7 staining in the normal kidney tissue was less dense and was observed only in the cytoplasm. Additionally, MMP-7 staining was observed mainly in the kidney tubule epithelium. Compared with CCRCC tissue, normal tissue demonstrated a significantly lower percentage of positive staining for MMP-7 (28.6%, 8/28; P<0.01; Table II). This suggests that MMP-7 expression is significantly upregulated in CCRCC tissue. Furthermore, positive associations between MMP-7 expression and pathological grade (χ^2^=12.20, P<0.01) and clinical stage (χ^2^=8.44, P<0.05) were evident (Fig. 1E and F; Table III).

### TIMP-2 expression in CCRCC and normal kidney with immunohistochemical staining

A representative kidney section stained immunohistochemically for TIMP-2 is shown in Fig. 1C. In normal kidney tissue, TIMP-2 staining was observed in the cytoplasm of the kidney tubule epithelium (Fig. 1D and H), with a positive expression rate of 82.1% (23/28). By contrast, CCRCC tissue demonstrated clear TIMP-2 staining only in the cytoplasm of cancerous cells and this staining was less dense. Normal tissue underwent significantly more staining than CCRCC tissue (44.9%, 44/98; P<0.01; Table II). This suggests that in CCRCC tissue TIMP-2 expression is significantly downregulated. Furthermore, negative associations of TIMP-2 expression with pathological grade (χ^2^=7.98, P<0.05) and clinical stage (χ^2^=7.97, P<0.05) were evident (Fig. 1G; Table IV).

### Association between MMP-7 and TIMP-2 expression in CCRCC tissues

There was a statistically significant negative correlation between MMP-7 and TIMP-2 expression (r=-0.416, P<0.01; Table V).

### Association between MMP-7 and TIMP-2 expression in CCRCC tissues and prognosis in CCRCC patients

**Figure 2.**
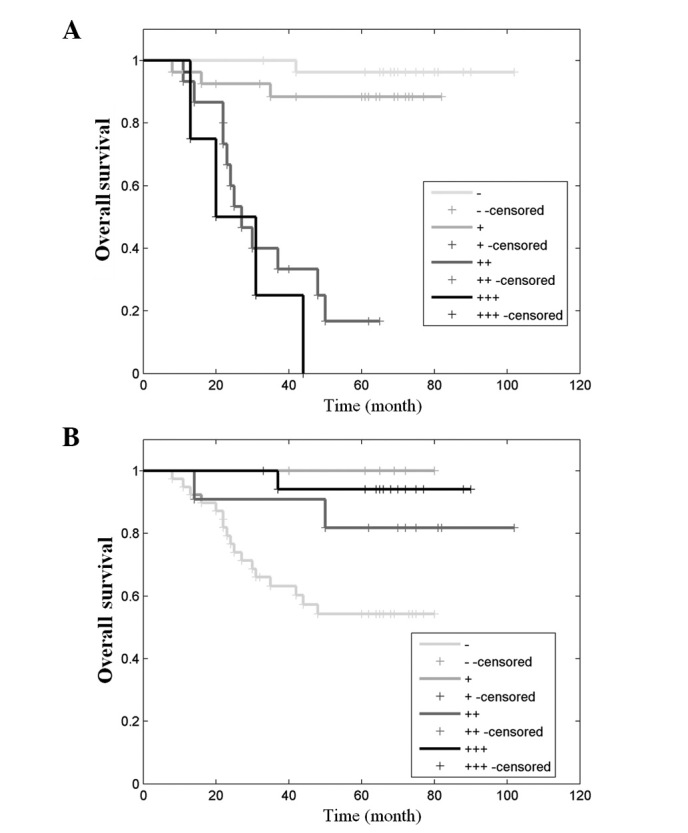
(A) Survival curves based on MMP-7 staining. (B) Survival curves based on TIMP-2 staining. MMP-7, matrix metalloproteinase 7; TIMP-2, tissue inhibitor of matrix metalloproteinases 2.

Seventy-four patients were subjected to a follow-up and their expected survival curves were calculated using the Kaplan-Meier method (Fig. 2). Survival curves were calculated based on MMP-7 and TIMP-2 expression levels. Significant negative associations between MMP-7 and TIMP-2 expression and survival rates were evident. The survival rate in the 5 years following tumor resection was estimated to be 95, 85, 20 and 0% for patients with -, +, ++ and +++ MMP-7 expression, respectively, and 50, 80, 93 and 100% for patients with -, +, ++ and +++ TIMP-2 expression, respectively. Patients with - and + MMP-7 or ++ and +++ TIMP-2 expression had a significantly longer expected survival time as compared with those with ++ and +++ MMP-7 or - and + TIMP-2 expression.

### Association between MMP-7 and TIMP-2 expression in CCRCC tissues and pathological grade, clinical stage and patient prognosis

Univariate survival rate analysis demonstrated a statistically significant association between patient prognosis and pathological grade, clinical stage, MMP-7 expression and TIMP-2 expression (P<0.01). However, no significant association was observed between patient prognosis and gender, age, tumor size or kidney vein cancer bolt (data not shown). A Cox model was used to analyze the correlation between survival rate and the four positive parameters mentioned above. This revealed pathological grade, clinical stage and MMP-7 expression as three independent factors negatively correlated with post-CCRCC expected survival rate (P<0.01, P<0.05 and P<0.05, respectively). Higher pathological grade, higher clinical stage and increased expression of MMP-7 correlated with a worse prognosis (Table VI).

### Expression of MMP-7 and TIMP-2 mRNA in CCRCC and normal kidney with RT-PCR

**Figure 3.**
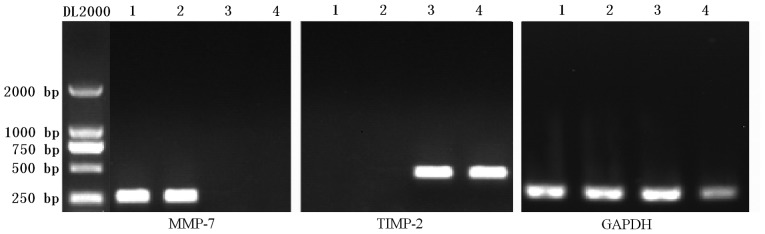
Expression of MMP-7 and TIMP-2 genes. RT-PCR was used to evaluate the expression of MMP-7, TIMP-2 and GAPDH mRNA in normal and CCRCC kidney tissues. Representative images of RT-PCR products are shown. Lanes 1 and 2 contain two CCRCC tissues whereas lanes 3 and 4 contain two normal kidney tissues. GAPDH products serve as internal controls for each case. MMP-7, matrix metalloproteinase 7; TIMP-2, tissue inhibitor of matrix metalloproteinases 2; RT-PCR, reverse transcription-polymerase chain reaction; GAPDH, glyceraldehyde 3-phosphate dehydrogenase; CCRCC, clear cell renal cell carcinoma.

By RT-PCR analysis we observed mRNA expression of the control gene GAPDH in normal and CCRCC tissue and observed no significant difference between the GAPDH expression levels in these two types of tissues (Fig. 3). An extremely weak expression of MMP-7 mRNA was measured in the CG and no expression of TIMP-2 mRNA was observed in CCRCC. However, there was a significant expression of MMP-7 in CCRCC and TIMP-2 mRNA in the CG.

### Association between MMP-7 gene expression in CCRCC tissue and pathological grade and clinical stage

**Figure 4.**
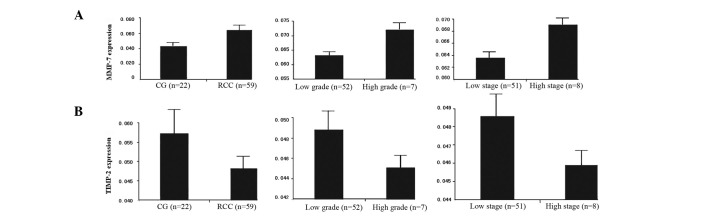
Levels of MMP-7 and TIMP-2 mRNA expression detected by quantitative RT-PCR in CCRCC and the control group. (A) Differential expression of MMP-7 mRNA in the control group and CCRCC; differential expression of MMP-7 mRNA in low and high grades and differential expression of MMP-7 mRNA in low and high stages. (B) Differential expression of TIMP-2 mRNA in the control group and CCRCC; differential expression of TIMP-2 mRNA in low and high grades; differential expression of TIMP-2 mRNA in low and high stages. MMP-7, matrix metalloproteinase 7; TIMP-2, tissue inhibitor of matrix metalloproteinases 2; RT-PCR, reverse transcription-polymerase chain reaction; CCRCC, clear cell renal cell carcinoma; CG, control group.

Among the 59 CCRCC cases for which RT-PCR analysis was performed, there was a significant increase in MMP-7 mRNA expression as compared with the 22 control samples. Among the CCRCC patients, as shown in Table VI, the expression of MMP-7 was significantly upregulated in the high grade CCRCC (grades III and IV) compared with the low grade CCRCC (grades I and II; P<0.05). There was also a significant difference in the positive expression of MMP-7 mRNA between cases with high clinical stage (stages III and IV) and low clinical stage (stages I and II; P<0.05; Fig. 4A).

### Association between TIMP-2 gene expression in CCRCC tissues and pathological grade and clinical stage

Although normal kidney and CCRCC tissues demonstrated TIMP-2 mRNA expression, quantitative analysis revealed a significantly lower level of expression of TIMP-2 mRNA in the CCRCC tissues, as compared with normal kidney tissue. Furthermore, there were significant associations between TIMP-2 mRNA expression and pathological grading and clinical staging. The expression of TIMP-2 mRNA was significantly higher in the low grades than in the high grades and in the low stages than in the high stages (Fig. 4B).

## Discussion

MMPs are enzymes produced by stromal or tumor cells and are involved in tumor progression. TIMPs are induced in stromal cells to regulate the proteinase reactions. They are closely related to a series of pathological processes. The imbalance between MMP and TIMP plays a critical role in cancer invasion and metastasis (20–22).

The role of MMPs and TIMPS in RCC growth, metastasis and angiogenesis has been the focus of intense investigation for a number of years. Awakura *et al* demonstrated by univariate analysis that TIMP-2 is a significant prognostic factor (1). Kawata *et al* identified that the expression of TIMP-2 has an essential correlation with the expression of MMP-2, which may have a correlation with the prognosis of CCRCC. Moreover, the authors indicated that nuclear grade and TIMP-2 are significant prognostic factors of CCRCC and that patients with tumors with a high pathological grade and strongly expressed MMP-9 and TIMP-2 have a poor outcome (23,24). Miyata *et al* reviewed tissue samples of 156 RCC patients and demonstrated that MMP-7 affects tumor progression by regulating invasion and angiogenesis and is a predictor of poor prognosis by multivariate analysis (25). However, another study reported that MMP-7 expression is not associated with clinicopathological features, including grade, invasion and metastasis in a number of cancers (26). Thus, there is a difference of opinion regarding the clinical significance of MMP-7 in cancers.

In the current study, we focused on the roles of MMP-7 and TIMP-2 in the tissue of 98 CCRCC patients in relation to the clinicopathological characteristics of tumors, including pathological grade and clinical stage, and the survival rate of the patients, using tissue microarray, immunohistochemistry and RT-PCR. The results of immunohistochemical analysis demonstrated that the expression level of MMP-7 in CCRCC was significantly higher than in the CG. Moreover, high MMP-7 expression was correlated with the degree of malignancy, including high grade and high stage. In addition, univariate survival rate analysis demonstrated that patients with an elevated expression of MMP-7 in the CCRCC tissue were predicted to have a poor prognosis. TIMP-2 expression level in CCRCC was clearly lower than in the CG and the high expression correlated with low grade and low stage. Univariate survival rate analysis demonstrated that, contrary to MMP-7 expression, patients with an elevated expression of TIMP-2 had a good survival rate. Correlation analysis revealed negative correlations between MMP-7 and TIMP-2 expression levels. Cox multivariate survival rate analysis demonstrated positive correlations between MMP-7 expression level and a high potential for tumor invasion and metastasis, and a poor prognosis. Through RT-PCR analysis, we also confirmed the high expression of MMP-7 and low expression of TIMP-2 in cases with high pathological grade and clinical stage.

These findings revealed that the expression levels of MMP-7 and TIMP-2 in CCRCC tissues are related to malignant progression in RCC and also to survival rate following tumor removal. Therefore, expression of these proteins may be considered indicators of progression in CCRCC tumors and prognostic predictors in CCRCC patients. These findings also indicate that MMP-7 is an independent prognostic factor but TIMP-2 is not, which differs from the conclusions of other studies (23,24,26). Thus, MMP-7 and TIMP-2 may be useful molecular markers for evaluating prognosis in CCRCC patients.

In conclusion, we demonstrated for the first time that MMP-7 is associated with TIMP-2 expression in CCRCC. The concentrations of MMP-7 and TIMP-2 in the tissue of 98 CCRCC patients were assessed in relation to pathological grade, clinical stage and survival rate. Upregulated expression of MMP-7 and downregulated expression of TIMP-2 in CCRCC have significant clinicopathological associations with the aggressiveness observed for this tumor. In addition, we identified that elevated levels of MMP-7 in cancer tissues are a strong and independent predictor of poor prognosis.

Therefore, MMP-7 and TIMP-2 may be useful molecular markers for evaluating prognosis in CCRCC patients and MMP-7 may be a new target for the prevention of tumor development and improvement of survival rate.

## Figures and Tables

**Table I.** MMP-7, TIMP-2 and GAPDH primer nucleotide sequences.

Gene name		Primer sequence (5′-3′)	Product length (bp)
MMP-7	Sense	AGCCATGTATCAGAGTCACCAA	326
	Antisense	AGTATCAGGAGCAGGAGAG	
TIMP-2	Sense	GGGGTTTTGGAATGCAGATGTAG	418
	Antisense	CACAGGAGCCGTCACTTCTCTTG	
GAPDH	Sense	GGATATCGCATCACCATCT	287
	Antisense	GAGTGCTTTCACGATACCAA	

MMP-7, matrix metalloproteinase 7; TIMP-2, tissue inhibitor of matrix metalloproteinases 2; GAPDH, glyceraldehyde 3-phosphate dehydrogenase.

**Table II.** Expression of MMP-7 and TIMP-2 in the CCRCC and control groups.

		MMP-7				TIMP-2			
Group	n	+ - +++	%	χ^2^	P-value	+ - +++	%	χ^2^	P-value
CG	28	8	28.6			23	82.1		
CCRCC	98	61	62.2	9.97	<0.01	44	44.9	12.13	0.01

MMP-7, matrix metalloproteinase 7; TIMP-2, tissue inhibitor of matrix metalloproteinases 2; CCRCC, clear cell renal cell carcinoma; CG, control group.

**Table III.** MMP-7 expression, pathological grade and clinical stage of CCRCC.

	MMP-7				
Case	N	-	+	++	+++
Pathological grade					
I	47	26	15	6	0
II	39	10	21	7	1
III	8	1	2	3	2
IV	4	0	0	1	3
Clinical stage					
I	61	29	23	9	0
II	24	7	13	3	1
III	8	1	2	3	2
IV	5	0	0	2	3

MMP-7, matrix metalloproteinase 7; CCRCC, clear cell renal cell carcinoma.

**Table IV.** TIMP-2 expression, pathological grade and clinical stage of CCRCC.

	TIMP-2				
Case	N	-	+	++	+++
Pathological grade					
I	47	22	5	9	11
II	39	21	4	6	8
III	8	7	1	0	0
IV	4	4	0	0	0
Clinical stage					
I	1	28	6	12	15
II	24	15	2	3	4
III	8	6	2	0	0
IV	5	5	0	0	0

TIMP-2, tissue inhibitor of matrix metalloproteinases 2; CCRCC, clear cell renal cell carcinoma.

**Table V.** Expression of MMP-7 associated with TIMP-2 in CCRCC.

	TIMP-2				
MMP-7	-	+	++	+++	Total
-	9	6	8	14	37
+	28	2	5	3	38
++	12	1	2	2	17
+++	5	1	0	0	6
Total	54	10	15	19	98

MMP-7, matrix metalloproteinase 7; TIMP-2, tissue inhibitor of matrix metalloproteinases 2; CCRCC, clear cell renal cell carcinoma.

**Table VI.** Multiple-factor Cox model regression variables of CCRCC.

	95% CI for HR			
	P-value	HR	Lower	Upper
MMP-7	0.023	1.650	0.871	3.12
TIMP-2	0.320	0.741	0.410	1.338
Pathological grade	0.005	2.842	1.370	5.896
Clinical stage	0.011	1.650	1.178	3.697

CCRCC, clear cell renal cell carcinoma; MMP-7, matrix metalloproteinase 7; TIMP-2, tissue inhibitor of matrix metalloproteinases 2; HR, hazard ratio; CI, confidence interval.
